# Supplementary material for: Smooth Interpolating Curves with Local Control and Monotone Alternating Curvature
Source: Comput Graph Forum. 2022 Oct 6;41(5):25–38. doi: 10.1111/cgf.14600 (PMC9827861; doi:10.1111/cgf.14600)
Supplement: Supplementary file 1 — Supplement Material [file CGF-41-25-s001.zip › Local-Smooth-Interpolating-MonoCurvature/extern/clothoids/submodules/quarticRootsFlocke/docs/api-cpp/unabridged_api.html]

Full API — Quartic Roots v1.0.0

### Navigation

- index
- toc
- Quartic Roots »
- Full API

# Full API¶

## Namespaces¶

- Namespace PolynomialRoots
  - Detailed Description
  - Classes
  - Functions
  - Typedefs

## Classes and Structs¶

- Class Cubic
  - Class Documentation

- Class Quadratic
  - Class Documentation

- Class Quartic
  - Class Documentation

## Functions¶

- Function PolynomialRoots::roots
  - Function Documentation

## Typedefs¶

- Typedef PolynomialRoots::complexType
  - Typedef Documentation

- Typedef PolynomialRoots::indexType
  - Typedef Documentation

- Typedef PolynomialRoots::valueType
  - Typedef Documentation

### Quick search

### Table of Contents

- Quartic Roots Solver
- C++ API

«
hide menu

menu
sidebar
»

### Navigation

- index
- toc
- Quartic Roots »
- Full API

© Copyright 2021, Enrico Bertolazzi.
Created using Sphinx 4.2.0.
